# Image Reconstruction of a Charge Coupled Device Based Optical Tomographic Instrumentation System for Particle Sizing

**DOI:** 10.3390/s101009512

**Published:** 2010-10-22

**Authors:** Mariani Idroas, Ruzairi Abdul Rahim, Robert Garnet Green, Muhammad Nasir Ibrahim, Mohd Hafiz Fazalul Rahiman

**Affiliations:** 1 Faculty of Chemical & Natural Resources Engineering, University Teknologi Malaysia, 81310 UTM Skudai, Johor, Malaysia; 2 Faculty of Electrical Engineering, University Teknologi Malaysia, 81310 UTM Skudai, Johor, Malaysia; 3 School of Engineering, Sheffield Hallam University, Sheffield, UK; 4 School of Mechatronic Engineering, University Malaysia Perlis, 02600 Arau, Perlis, Malaysia

**Keywords:** bubbles velocity, optical tomography, inverse algorithm

## Abstract

This research investigates the use of charge coupled device (abbreviated as CCD) linear image sensors in an optical tomographic instrumentation system used for sizing particles. The measurement system, consisting of four CCD linear image sensors are configured around an octagonal shaped flow pipe for a four projections system is explained. The four linear image sensors provide 2,048 pixel imaging with a pixel size of 14 micron × 14 micron, hence constituting a high-resolution system. Image reconstruction for a four-projection optical tomography system is also discussed, where a simple optical model is used to relate attenuation due to variations in optical density, [*R*], within the measurement section. Expressed in matrix form this represents the forward problem in tomography [*S*] [*R*] = [*M*]. In practice, measurements [*M*] are used to estimate the optical density distribution by solving the inverse problem [*R*] = [*S*]^−1^[*M*]. Direct inversion of the sensitivity matrix, [*S*], is not possible and two approximations are considered and compared—the transpose and the pseudo inverse sensitivity matrices.

## 1. Process Tomography

Industrial processes are often controlled using process measurements at one or more points. The amount of information contained in such measurements is often minimal, and in some cases (multiphase flow) there are no adequate sensors [1]. To understand better certain chemical processes, a more sophisticated approach is needed. Process tomography is a means of visualising the internal behaviour of industrial processes, where tomographic images provide valuable information about the process for assessment of equipment designs and on-line monitoring [2,3].

There are several modalities used in process tomography such as electrical (impedance, capacitance, inductance), radiation (optical, x-ray, positron electromagnetic (PET), magnetic resonance) and acoustic (ultrasonic) [4,5]. Electrical tomography has relatively poor spatial resolution of about 10% of diameter of cross section [6]. The X-ray computed tomography method is well known, but specific safety procedures need to be followed by the operator. PET needs operator intervention and radioactive particles. Ultrasonic tomography is complex to use due to spurious reflections and diffraction effects and may therefore require a high degree of engineering design [4].

Optical techniques are desirable because of their inherent safety (the transducer does not require direct physical contact with the measurand), high efficiency [7] and could improve manufacturing in the chemical industries [8]. For processes handling transparent fluids and where optical access is possible, optical techniques can provide high-resolution images [2] *i.e.*, 1% spatial resolution [9].

**Figure 1.**
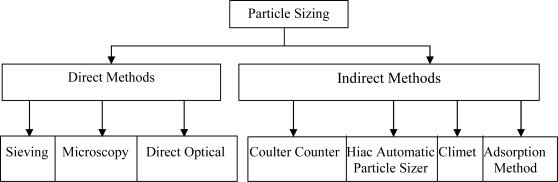
A block diagram of particle sizing techniques.

Particle sizing is very important for many industrial processes and has led to much research. Typical problems relate to pulverised coal for combustion and liquid fuels, spray characterisations, analysis and control of particulate emissions, industrial process control, manufacture of metallic powders and the production of pharmaceuticals [10,11]. Figure 1 summarises the major techniques used in particle-size measurements [12].

The majority of these techniques are off-line, with direct optical providing the only truly on-line measurement [10]. The existing on-line optical methods use Fraunhofer diffraction to determine positions or angles of optical emission spectra generally within a limited measurement volume, which sets a limit on the quality of images produced by optical systems [13]. Morikita *et al.* [14] used the Fraunhofer response curve (size-intensity relationship) to measure equivalent diameter of non-spherical particles ranging from 20–200 μm; both these methods are inferential.

Horbury *et al* [15] investigated transparent slurries with particle sizing and flow profiles using optical fibres. An optical tomography system that uses optical fibre bundles has problems in ensuring every fibre has similar optical characteristics [16]. Thus, a system based on CCD devices is proposed which may provide very high resolution (better than 1%), on-line measurement over the full measurement cross section and high speed data acquisition based on proprietary items [17].

## 2. The Optical System

**Figure 2.**
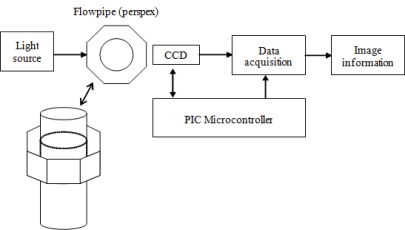
A block diagram of the optical tomography system.

Mathematical modelling is an important tool in simulating a system. It can predict the output of a system with known conditions. In addition, the model enables the user to understand the output trends or behaviour. Three types of mathematical model are developed in this project for investigating the effects due to particles, the effects due to light sources and the effects due to diffraction on the optical tomography system. The complete optical tomography system consists of a lighting system, the measurement section, the sensor system, the data acquisition system, the PIC microcontroller system and image information. Figure 2 shows an overview of the system.

A well-collimated beam of light is passed through the measurement section, past the object to be measured. For test and calibration purposes, aground bar of known diameter is used. The detection system is the sensing component in the optical tomography system. The standard object (ground bar of known diameter) is sensed using a charge coupled device (CCD) linear image sensor. The output of the CCD linear image sensor is then acquired by the data acquisition system.

## 3. Introduction to Image Reconstruction

Image reconstruction is a process of generating an image from raw data, or a set of unprocessed measurements, made by the imaging system. In general, there is a well-defined mathematical relationship between the distribution of physical properties in an object and the measurements made by the imaging system. Image reconstruction is the process which inverts this mathematical process to generate an image from the set of measurements [18].

In an optical imaging system, the object density along the optical path (according to Beer-Lambert Law) exponentially attenuates the light intensity:

(1) $$I_{\mathrm{out}}=I_{\mathrm{in}}e^{-\alpha x}$$

(2) $$\text{ln}\left(\frac{I_{\mathrm{in}}}{I_{\mathrm{out}}}\right)=\alpha x$$

where α is the linear attenuation coefficient and x is the distance the light traverses. The natural logarithm of the ratio of the incident intensity to the transmitted intensity is equal to the line integral or ray sum of the distribution of linear attenuation coefficients within the object along the path. An image of the object density distribution can be created using a projection reconstruction algorithm.

The optical tomography system consists of four projection systems where each projection is generated by a ray-box which has a laser diode, objective lens, and an spherical lens in it and a CCD linear image sensor at the other side of the pipe. The CCD linear image sensor used in the system has 2,048 effective pixels with a pixel size of 14 micron by 14 micron.

As a result, the tomographic image consists of four projections, with each providing 2,048 measurements. The complete forward problem requires 2,048 × 2,048 values of α (attenuation coefficient) to model the system. The image reconstruction process for the system is modelled using smaller *m* by *n* arrays of pixels to make the problem solvable using a personal computer (PC). This approach reduces the processing time and enables a better understanding of the impact of each technique when applied to the actual system.

**Figure 3.**
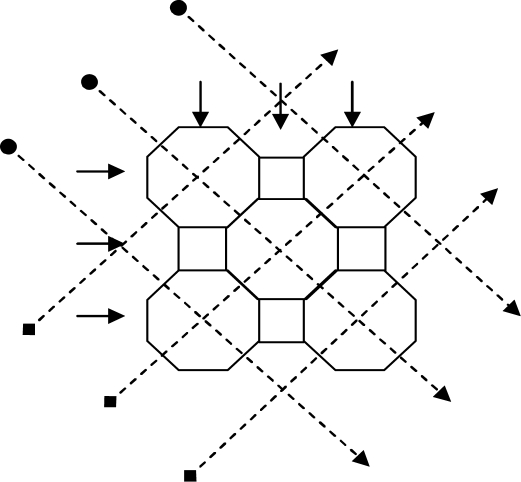
Four projections on 3 × 3 cells.

To explain the forward problem simply the object space is considered to consist of a 7 × 7 array of cells projected onto a circular measurement cross-section, later this is extended to the 2,048 × 2,048 array. The analysis is for four projections (Figure 3). For the inverse problem several different arrangements are investigated. These include three projections on a 3 × 3 array of cells, four projections on a 4 × 4 array cells and two, three and four projections on a 7 × 7 array of cells. This analysis aims to compare the effects of using pseudo inverse and transpose of the sensitivity matrix.

A simple optical attenuation model is used to model the measurement system. The model uses an array of octagonal shaped cells with square cells to ensure that the light traverses through all cells normally (Figure 3). Moreover, the arrangement of cells based on the octagon and square shape simplify the matrix manipulation in the image reconstruction process. The light beam width is assumed to be 0.006 mm *i.e.*, passes through the square cell and the octagonal cell normally. In this case, the octagonal cell has four projections whilst the square cell has only two projections. However, the effect of the pixel shapes on the reconstructed image is not significant in the actual system due to its large number of pixels (2,048 × 2,048 pixels) and small particles of interest (100 micron up to 10 mm diameter particles).

**Figure 4.**
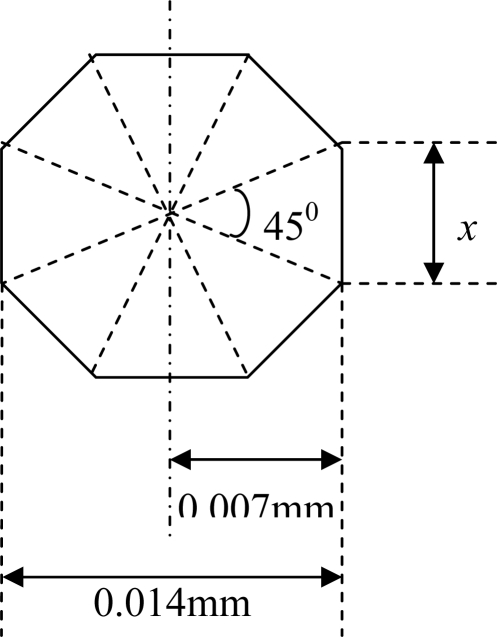
An octagonal shaped cell.

The dimension of each side of the octagonal cell is calculated as follows (refer to Figure 4):

(3) (tan⁡ 22.5°)=(x/2)/(0.007)

(4) x=0.014 (tan⁡ 22.5°)=0.0058 mm≈0.006 mm

**Figure 5.**
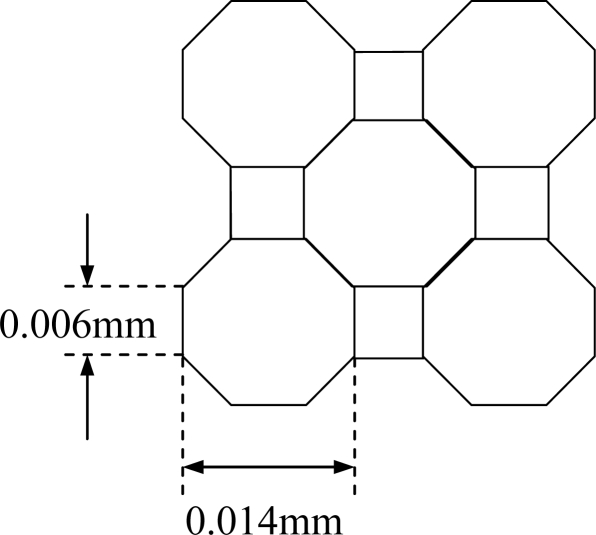
Actual measurements of octagonal-square shaped cells.

The actual measurement of the octagonal and square cells is shown in Figure 5.

**Figure 6.**
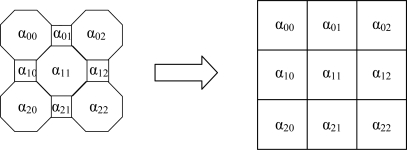
Transformation of octagonal-square cells into square image pixels.

The optical attenuation model (based on the combination of octagonal-square shaped cells) is used in the forward problem. These cells are used in the formation of the sensitivity matrix for the image reconstruction process. However, the reconstructed image from the inverse problem has square image pixels due to the standard format of a matrix. The pixel size is 0.014 mm by 0.014 mm for the actual reconstructed image system (2,048 × 2,048 array). The arrangement of cells is shown in Figure 6.

## 4. Image Reconstruction Process

Several techniques have been used in optical tomography to produce images from measured data, such as layergram back-projection (LYGBP) by Ibrahim [19], linear back-projection (LBP) by Abdul Rahim [7], Algebraic reconstruction technique (ART) by Reinecke and Mewes [19], iteration techniques, Fourier inversion techniques and others [20].

Xie [6] highlighted techniques used in transmission tomography such as optical and X-ray methods, which are based on straight-line propagation. The forward problem has to be performed first in order to obtain the expected output from the sensors (for known attenuation coefficients of water and the particle). The calculated output from the forward problem is then used in the back-projection process—the inverse problem.

### 4.1. Forward Problem

**Figure 7.**
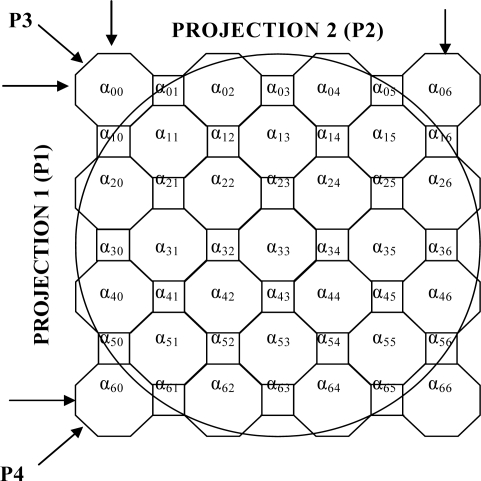
7 × 7 array of pixels with four projections.

In this project, the particle may be translucent or opaque and is a solid surrounded by water. The particle used in the modelling analysis is considered to have a linear attenuation coefficient of 10 mm^−1^. To explain the forward problem process, the area to be imaged in the pipe is divided into 49 cells (a 7 × 7 array of cells) for simplicity. Later in this section the model is extended to a 2,048 × 2,048 array of cells. The reconstructed image of the particle in water is based on four projections. The arrangement of cells is shown in Figure 7.

From Figure 7 each projection has seven optical sensors and hence the total number of sensors for four projections (P1 to P4) is twenty-eight which provide twenty-eight measurements—M1 to M28. There are up to seven cells associated with each sensor when the incoming light passes through the pipe.

The linear attenuation of the light is modelled by assuming that each cell has an attenuation coefficient of α_ij_ where i and j represent the row and column, respectively. The length of the octagon pixel is 0.014 mm whilst the length of the square pixel is 0.006 mm. The change in light intensity measured by the sensors may be written as *ln (Constant/I_out_)_n_* = *M_n_* where *n* = 1 to 28

Based on the Beer-Lambert Law of absorption (Equations 1 and 2), the horizontal equations (*i.e.*, projection 1) for each sensor are (Equations 5 to 11):

$$\alpha_{00}(0.014)+\alpha_{01}(0.006)+\alpha_{02}(0.014)+\alpha_{03}(0.006)+\alpha_{04}(0.014)+\alpha_{05}(0.006)+\alpha_{06}(0.014)=M_{1}$$

$$\alpha_{10}(0.006)+\alpha_{11}(0.014)+\alpha_{12}(0.006)+\alpha_{13}(0.014)+\alpha_{14}(0.006)+\alpha_{15}(0.014)+\alpha_{16}(0.006)=M_{2}$$

$$\alpha_{20}(0.014)+\alpha_{21}(0.006)+\alpha_{22}(0.014)+\alpha_{23}(0.006)+\alpha_{24}(0.014)+\alpha_{25}(0.006)+\alpha_{26}(0.014)=M_{3}$$

$$\alpha_{30}(0.006)+\alpha_{31}(0.014)+\alpha_{32}(0.006)+\alpha_{33}(0.014)+\alpha_{34}(0.006)+\alpha_{35}(0.014)+\alpha_{36}(0.006)=M_{4}$$

$$\alpha_{40}(0.014)+\alpha_{41}(0.006)+\alpha_{42}(0.014)+\alpha_{43}(0.006)+\alpha_{44}(0.014)+\alpha_{45}(0.006)+\alpha_{46}(0.014)=M_{5}$$

$$\alpha_{50}(0.014)+\alpha_{51}(0.006)+\alpha_{52}(0.014)+\alpha_{53}(0.006)+\alpha_{54}(0.014)+\alpha_{55}(0.006)+\alpha_{56}(0.014)=M_{6}$$

$$\alpha_{60}(0.014)+\alpha_{61}(0.006)+\alpha_{62}(0.014)+\alpha_{63}(0.006)+\alpha_{64}(0.014)+\alpha_{65}(0.006)+\alpha_{66}(0.014)=M_{7}$$

Similarly, for the vertical equations (projection 2) for each sensor (Equations 12 to 18):

$$\alpha_{00}(0.014)+\alpha_{10}(0.006)+\alpha_{20}(0.014)+\alpha_{30}(0.006)+\alpha_{40}(0.014)+\alpha_{50}(0.006)+\alpha_{60}(0.014)=M_{8}$$

$$\alpha_{01}(0.006)+\alpha_{11}(0.014)+\alpha_{21}(0.006)+\alpha_{31}(0.014)+\alpha_{41}(0.006)+\alpha_{51}(0.014)+\alpha_{61}(0.006)=M_{9}$$

$$\alpha_{02}(0.006)+\alpha_{12}(0.014)+\alpha_{22}(0.006)+\alpha_{32}(0.014)+\alpha_{42}(0.006)+\alpha_{52}(0.014)+\alpha_{62}(0.006)=M_{10}$$

$$\alpha_{03}(0.006)+\alpha_{13}(0.014)+\alpha_{23}(0.006)+\alpha_{33}(0.014)+\alpha_{43}(0.006)+\alpha_{53}(0.014)+\alpha_{63}(0.006)=M_{11}$$

$$\alpha_{04}(0.006)+\alpha_{14}(0.014)+\alpha_{24}(0.006)+\alpha_{34}(0.014)+\alpha_{44}(0.006)+\alpha_{54}(0.014)+\alpha_{64}(0.006)=M_{12}$$

$$\alpha_{05}(0.006)+\alpha_{15}(0.014)+\alpha_{25}(0.006)+\alpha_{35}(0.014)+\alpha_{45}(0.006)+\alpha_{55}(0.014)+\alpha_{65}(0.006)=M_{13}$$

$$\alpha_{06}\left(0.006\right)+\alpha_{16}\left(0.014\right)+\alpha_{26}\left(0.006\right)+\alpha_{36}\left(0.014\right)+\alpha_{46}\left(0.006\right)+\alpha_{56}\left(0.014\right)+\alpha_{66}\left(0.006\right)=M_{14}$$

Projection 3 (Equations 19 to 25):

$$\alpha_{06}\left(0.014\right)\;=M_{15}$$

$$\alpha_{04}\left(0.014\right)+\alpha_{15}\left(0.014\right)+\alpha_{26}\left(0.014\right)=M_{16}$$

$$\alpha_{02}\left(0.014\right)+\alpha_{13}\left(0.014\right)+\alpha_{24}\left(0.014\right)+\alpha_{35}\left(0.014\right)+\alpha_{46}\left(0.014\right)\;=M_{17}$$

$$\alpha_{00}\left(0.014\right)+\alpha_{11}\left(0.014\right)+\alpha_{22}\left(0.014\right)+\alpha_{33}\left(0.014\right)+\alpha_{44}\left(0.014\right)+\alpha_{55}\left(0.014\right)+\alpha_{66}\left(0.014\right)=M_{18}$$

$$\alpha_{20}\left(0.014\right)+\alpha_{31}\left(0.014\right)+\alpha_{42}\left(0.014\right)+\alpha_{53}\left(0.014\right)+\alpha_{64}\left(0.014\right)\;=M_{19}$$

$$\alpha_{40}\left(0.014\right)+\alpha_{51}\left(0.014\right)+\alpha_{62}\left(0.014\right)\;=M_{20}$$

$$\alpha_{60}\left(0.014\right)=M_{21}$$

Projection 4 (Equations 26 to 32):

$$\alpha_{66}(0.014)=M_{22}$$

$$\alpha_{64}(0.014)+\alpha_{55}(0.014)+\alpha_{46}(0.014)=M_{23}$$

$$\alpha_{62}(0.014)+\alpha_{53}(0.014)+\alpha_{44}(0.014)+\alpha_{35}(0.014)+\alpha_{26}(0.014)=M_{24}$$

$$\alpha_{60}(0.014)+\alpha_{51}(0.014)+\alpha_{42}(0.014)+\alpha_{33}(0.014)+\alpha_{24}(0.014)+\alpha_{15}(0.014)+\alpha_{06}(0.014)=M_{25}$$

$$\alpha_{40}(0.014)+\alpha_{31}(0.014)+\alpha_{22}(0.014)+\alpha_{13}(0.014)+\alpha_{04}(0.014)=M_{26}$$

$$\alpha_{20}(0.014)+\alpha_{11}(0.014)+\alpha_{01}(0.014)=M_{27}$$

$$\alpha_{00}(0.014)=M_{28}$$

These equations may be written in matrix form. The matrices shown above can be represented in a form of matrix [*S*] and matrix [*R*]:

(33) [S][R]=[M]

where [*S*] is the sensitivity matrix [28 × 49], [*R*] is the matrix of optical attenuation coefficients [49 × 1] and [*M*] is the measurement values [28 × 1]. This approach is now outlined for the full system.

**Figure 8.**
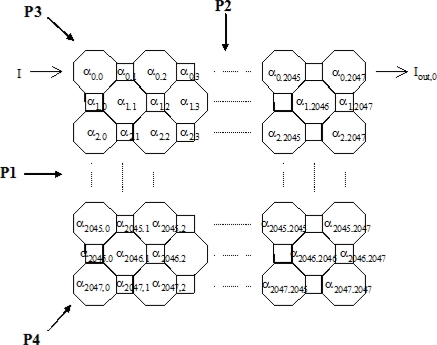
2,048 by 2,048 array of pixels with four projections.

A similar process is performed on the actual tomography system with 2,048 horizontal and 2,048 vertical cell (2,048 × 2,048 array). A simplified diagram of the cell attenuation coefficients is shown in Figure 8.

The equations for each of the projections for the 2,048 × 2,048 array (Figure 8)—projection 1, 2, 3 and 4 can be expressed in a similar manner to those shown in full for the 7 × 7 array.

Projection 1:
α_0,0_ a + α_0,1_ b + α_0,2_ a + ……………..α_0,2045_ a + α_0,2046_ b + α_0,2047_ a = ln (I/I_out,0_)
α_1,0_ a + α_1,1_ b + α_1,2_ a + ……………..α_1,2045_ a + α_1,2046_ b + α_1,2047_ a = ln (I/I_out,1_)
and so forth until:
α_2047,0_ a + α_2047,1_ b + α_2047,2_ a + …….α_2047,2045_ a + α_2047,2046_ b + α_2047,2047_ a = ln (I/I_out,0_)

Similarly for the vertical equations (projection 2):

α_0,0_ a + α_1,0_ b + α_2,0_ a + ……………..α_2045,0_ a + α_2046,0_ b + α_2047,0_ a = ln (I/I_out,2048_)
α_0,1_ a + α_1,1_ b + α_2,1_ a + ……………..α_2045,1_ a + α_2046,1_ b + α_2047,1_ a = ln (I/I_out,2049_)
and so forth until:
α_0,2047_ a + α_1,2047_ b + α_2,2047_ a + …….α_2045,2047_ a + α_2046,2047_ b + α_2047,2047_ a = ln (I/I_out,4095_)
Projection 3:
α_0,2047_ a = ln (I/I_out,4096_)
(α_0,2045_ + α_1,2046_ + α_2,2047_)*a* = *ln (I/I_out,4097_)*
and continue until:
α_2047,0_ *a* = *ln (I/I_out,6143_)*
Projection 4:
α_2047,2047_ a = ln (I/I_out,6144_)
(α_2047,2045_ + α_2046,2046_ + α_2045,2047_)a = ln (I/I_out,6145_)
and the same process is repeated until *α_0,0_* *a = ln (I/I_out,8191_)*

The above expressions can be expressed in matrix form,

(34) [S]×[R]=[M]

where [*S*] is the sensitivity matrix [8,192 × 4,194,304], [*R*] is the matrix of attenuation coefficients [4,194,304 × 1], and [*M*] is the measurement values [8,192 × 1].

### 4.2. Inverse Problem

In practice, measurements are made and then used to estimate values of the linear attenuation coefficients. Equation (5.34) needs to be re-arranged in order to reconstruct the tomographic image:

(35) $$\left[R\right]={\left[S\right]}^{-1}\times \left[M\right]$$

The main problems associated with the above equation are that the matrix [*S*] is not square, hence there is no direct inverse and it is also sparse (many of its values are zero). These are the main limitation of the inverse problem because it is not possible to have the number of projections equal to the number of cells involved. Even if the matrix [*S*] is square, it will be shown later that the inversion is still not possible because the matrix is too sparse. Many references such as Yang *et al* [21], Xie *et al* [22], Isaksen and Nordtvedt [23] and Salkeld [24] use the transpose of the sensitivity matrix to obtain qualitative information about the permittivity coefficients in Electrical Capacitance Tomography (ECT) measurements. A mathematically rigorous approach is to use the pseudo inverse of the matrix. Therefore, both the transpose and the pseudo-inverse are investigated in order to obtain an estimate of the inverse matrix of S:

(36) $$\left[R\right]={\left[S\right]}^{\mathrm{transpose}}\times \left[M\right]={\left[S\right]}^{T}\times \left[M\right]$$

(37) $$\left[R\right]={\left[S\right]}^{\mathrm{pseudo}-\mathrm{inverse}}\times \left[M\right]=\mathrm{pinv}\left[S\right]\times \left[M\right]$$

The values of matrix [*M*] are determined from the relationship between the intensity and the output voltage of the CCD linear sensor. The computation of matrix [*R*] (based on equations 5.36 and 5.37), is performed using Matlab software version 6.1. The values of matrix [*R*], which represent the optical attenuation coefficient, are then re-written as a square matrix. These values are represented by a colour in a graph. This graph is interpreted as a tomographic image with pixels of known size, and hence the physical size of the object can be obtained.

The following example shows the 2 × 2 array of numbers used to investigate the relationship between inverse, transpose, and pseudo-inverse of a matrix:

$$\begin{array}{ccc} & \begin{array}{cc} A=\left[\begin{array}{cc} 1 & 2\\ 3 & 4 \end{array}\right], & B=\left[\begin{array}{c} 1\\ 1 \end{array}\right] \end{array} & \\ A^{-1}=\left[\begin{array}{cc} -2 & 1\\ 1.5 & -0.5 \end{array}\right], & A^{T}=\left[\begin{array}{cc} 1 & 3\\ 2 & 4 \end{array}\right], & \mathrm{pinv}\left(A\right)=\left[\begin{array}{cc} -2 & 1\\ 1.5 & -0.5 \end{array}\right]\\ A^{-1}xB=\left[\begin{array}{c} -1\\ 1 \end{array}\right], & A^{T}xB=\left[\begin{array}{c} 4\\ 6 \end{array}\right], & \mathrm{pinv}(A)xB=\left[\begin{array}{c} -1\\ 1 \end{array}\right] \end{array}$$

The arguments show that pseudo-inverse in this case is equivalent to inversion of the matrix. In this case of matrix-inversion, the pseudo-inverse correctly represents the inverse matrix. However, the transpose does not give the correct answer. The transpose and pseudo inverse transforms are now used to generate images of several different models. The transpose is widely used in many tomographic researches due to its popularity, fast operation and simplicity (in terms of having the required dimensional form) [21].

## 5. Results

### 5.1. 3 × 3 Array of Pixels (Three Projections)

**Figure 9.**
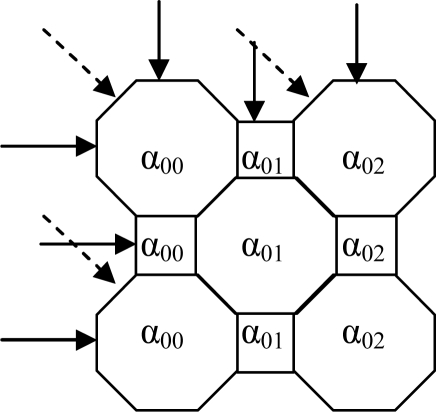
A 3 × 3 array of cells with three projections.

Figure 9 shows the arrangement of cells for a 3 × 3 array with three projections. In this case, the matrix S is square because the three projections give the same number of sensors (M_1_ to M_9_) as the test cells (nine cells of octagonal and square shapes). However, there is no inverse matrix of S because the matrix is too sparse.

**Figure 10.**
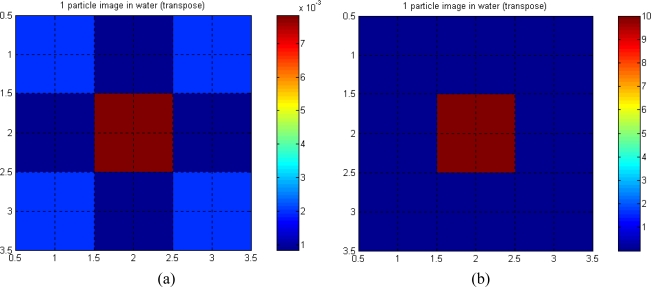
One particle in water. **(a)**.Transpose. **(b)**. Pseudo-inverse.

Figure 10(a,b) show the reconstructed image of a semi-opaque particle in water (in the middle), using transpose and pseudo-inverse matrices, respectively. The linear attenuation coefficient for the semi-opaque particle is assumed to be 10 mm^−1^, whilst for water it is 0.00287 mm^−1^.

**Figure 11.**
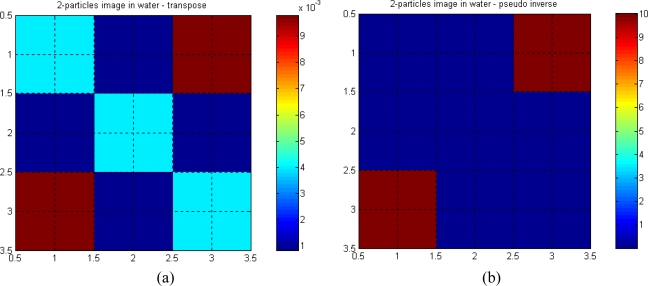
Two-particles in water. **(a)**. Transpose. **(b)**. Pseudo-inverse.

Figure 11(a,b) show reconstructed images when two similar particles are present in the measurement section.

The reconstructed images using the pseudo-inverse matrix are found to be better in representing the actual value of the linear attenuations of water and the particle, compared to the images using the transpose matrix. This is expected, as the pseudo-inverse matrix is closer to the matrix inversion when compared to the transpose matrix. Hence, the contrast is better in the pseudo-inverse image compared to the transpose image. The pseudo inverse image shows no indication of *aliasing* in the top left and bottom right hand corners of the image.

### 5.2. 7 × 7 Array of Pixels

In this section, three different projections are discussed—two, three and four projections. It is seen that the reconstructed image quality improves as the number of projections is increased.

**Figure 12.**
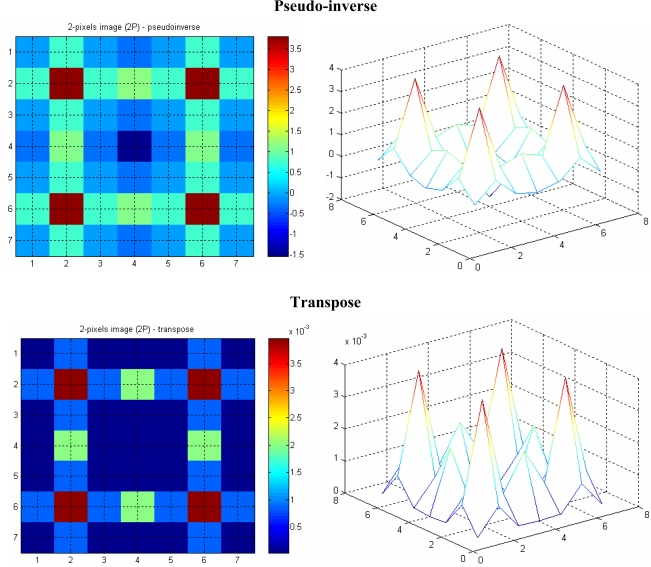
Two-particles image with two projections.

**Figure 13.**
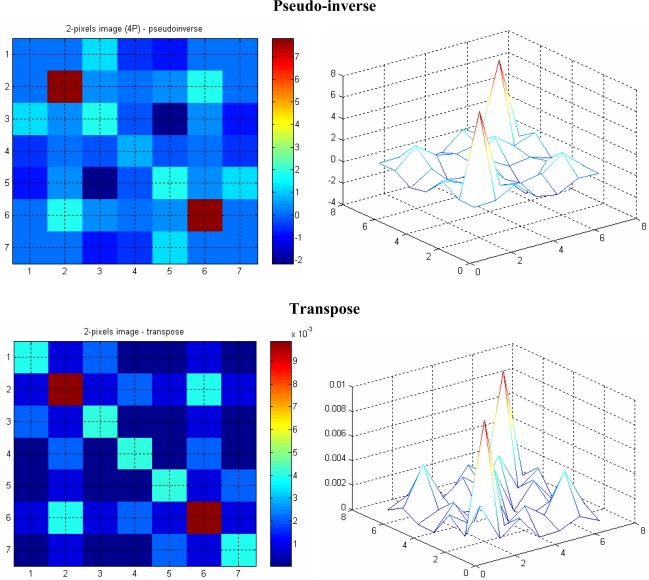
Two-particles image with four projections.

The image with four projections is less smeared, especially when the image is reconstructed using the pseudo-inverse matrix, compared to the one with two projections. Moreover, the effect of *aliasing* on the image is also reduced as the number of projections is increased. This is more noticeable when the image in Figure 12 (with two projections) is compared with the four-projection image in Figure 13.

### 5.3. 21 × 21 Array of Pixels

In the actual system, the effective cells to represent an object vary between approximately 7 cells (for a 100 micron particle) up to 71 cells (for a 1 mm particle). Since the optical tomography system is intended for small diameter particles, only a few of the whole 2,048 pixels are used. Hence, the reconstruction may be simplified by only imaging a small area of the measurement section.

**Figure 14.**
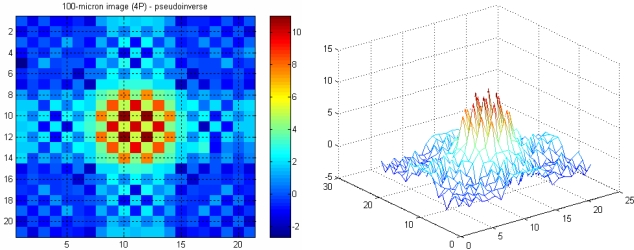
A 100 micron reconstructed image using pseudo inverse.

**Figure 15.**
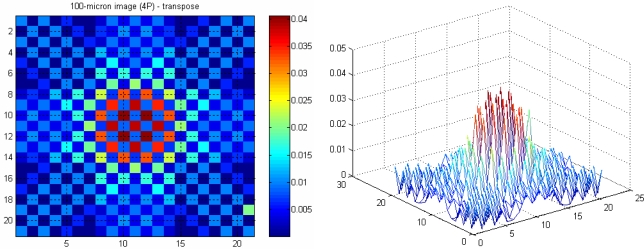
A 100 micron reconstructed image using transpose.

A 21 × 21 array of pixels actually represents 0.294 × 0.294 mm^2^ image area with an image pixel size of 0.014 mm by 0.014 mm. A 100 micron object with a round shape in water is modelled using the forward problem. The object is positioned in the middle of the measurement section, where the attenuation coefficients of water fill image pixels beyond this 0.294 × 0.294 mm^2^ image area. Figures 14 and 15 show images representing one 100 micron particle in water obtained using the pseudo inverse and the transpose matrices, respectively.

### 5.4. 101 × 101 Array of Pixels

In the actual system, the effective number of sensors per projection used to generate the image ranges from approximately 7 pixels (for a 100 micron particle) up to 71 pixels (for a 1 mm particle). Since the optical tomography system is intended for measuring small diameter particles, only about 5% (101 pixel/2,048 pixel × 100) of the whole 2,048 pixels per projection are used. Hence, a smaller array of pixels is used so that the reconstruction process is less complex (due to the smaller size of the matrices involved) and faster. In the actual system a 101 × 101 array of pixels is used for the reconstruction of the tomographic images *i.e.*, 1,414 × 1,414 mm^2^ area of image.

**Figure 16.**
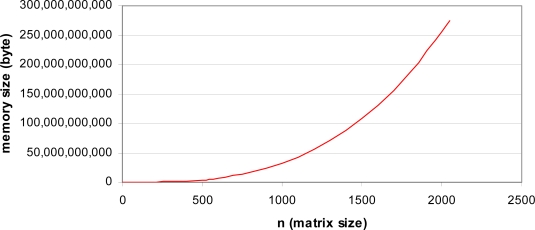
A graph of *n versus* memory size.

The full reconstruction operation requires a 2,048 × 2,048 array of pixels, which requires a lot of memory space (approximately 275 Gigabytes). The actual size of the matrix (four projections) = [4n by n^2^] where n is the number of sensors in each CCD linear image sensor. Therefore, the required memory size is the multiplication of the actual matrix size with 8 bytes *i.e.*, 32 n^3^. This result is only based on the amount of memory needed for the sensitivity matrix (S matrix) with four projections. The actual calculation would need more memory and hence the whole operation is just not possible on a PC. Figure 16 shows the minimum memory required to perform the reconstruction process as the numbers of pixels is increased.

The limitation of the computer memory limits the reconstructed image area to a 101 × 101 array of pixels (1,414 × 1,414 mm^2^). In order to reconstruct a larger object, a scaling process is done where several pixels are grouped together before averaging them. For example, averaging five CCD pixels to give a single measurement value which represents a spatial length of 0.07-mm. If the 101 × 101 array of pixels is used combined with this averaging, the image area is increased to 7.07 × 7.07 mm^2^.

## 6. Conclusions

A novel arrangement of cells with a combination of square-shaped cells and octagon-shaped cells makes the image reconstruction process possible for the proposed CCD-based optical tomography system. In this case, the square-shaped cell has two projections whereas the octagon-shaped cell has four projections of measurements. The tomographic images reconstructed using the transpose matrix is found to be producing qualitative images whilst the image reconstructed using the pseudo inverse matrix contains the quantitative information of the image. The main reason is that the pseudo inverse matrix represents the closest generalisation of matrix inversion. Moreover, the pseudo inverse minimises the error between the calculated values and the real values. On the other hand, the transpose calculates a value, which is often used as a basis for iterative image improvement. The transpose is much quicker to calculate than the pseudo inverse. Comparative times for a 101 × 101 pixel image are: 20 seconds (transpose) and 1 hour (pseudo inverse).
